# Antimicrobial Efficacy of an Ultraviolet-C Device against Microorganisms Related to Contact Lens Adverse Events

**DOI:** 10.3390/antibiotics11050699

**Published:** 2022-05-21

**Authors:** Srikanth Dumpati, Shehzad A. Naroo, Sunil Shah, Debarun Dutta

**Affiliations:** Optometry School, College of Health and Life Sciences, Aston University, Birmingham B4 7ET, UK; 200285764@aston.ac.uk (S.D.); s.a.naroo@aston.ac.uk (S.A.N.); s.shah26@aston.ac.uk (S.S.)

**Keywords:** contact lenses, ultraviolet C, keratitis, Pseudomonas, Staphylococcus, Fusarium, Candida, antibiotic resistance

## Abstract

The purpose of the study was to assess the antimicrobial activity of an ultraviolet-C (UVC) device against microorganisms implicated in contact lens related adverse events. An UVC device with an emitting 4.5 mm diameter Light Emitting Diode (LED; 265 nm; 1.93 mJ/cm^2^) was used. *Pseudomonas aeruginosa*, *Staphylococcus aureus*, *Fusarium solani*, and *Candida albicans* agar plate lawns were exposed to the device beams for 15 and 30 s at 8 mm distance. Following the exposure, the diameter of the growth inhibition zone was recorded. Contact lenses made of Delfilicon-A, Senofilicon-A, Comfilicon-A, Balafilicon-A, Samfilicon-A and Omafilicon-A and a commercially available contact storage case was used. They were exposed to bacterial and fungal strains for 18 h at 37 °C and 25 °C respectively. After this, the samples were exposed to UVC for 30 s at 8 mm distance to determine the antimicrobial efficacy. Samples were then gently washed and plated on appropriate agar for enumeration of colonies. The UVC exposure reduced microbial growth by 100% in agar lawns, and significantly (*p* < 0.05) reduced microbial contamination to contact lenses and cases, ranging between 0.90 to 4.6 log. Very short UVC exposure has high antimicrobial efficacy against most of the predominant causative microorganisms implicated in contact lens related keratitis. UVC could be readily used as a broad-spectrum antimicrobial treatment for lens disinfection.

## 1. Introduction

Contact lenses are an increasingly popular option for refractive correction with current estimates of more than 140 million wearers worldwide [1]. In addition, contact lenses are indispensable for patients with high astigmatism, high refractive error, irregular astigmatism, myopia control and are regularly used for post-surgical therapeutic use. However, contact lenses can be associated with various microbial adverse events such as microbial keratitis (MK), contact lens acute red eye (CLARE), contact lens peripheral ulcer (CLPU) and infiltrative keratitis (IK) [2]. 

MK is a worldwide medical concern often noted as the most serious form of contact lens infection, in the UK, 65% of all new cases of MK are contact lens-related [3]. The incidence of contact lens related-MK is around 4 per 10,000 a year for daily wear and 20 per 10,000 a year for extended wear [4]. Other less severe conditions have an even higher incidence whereby CLARE has been found to occur in up to 34% of those who regularly wear hydrogel contact lenses [5]. Sixty-six percent of complications observed in contact lens wearers are attributed to poor handling of lenses and lens cases [6]. Despite the introduction of silicone hydrogel materials, advancement in care products and cleaning regimens, the incidence of contact lens-related microbial adverse events remained unchanged [7]. The emergence of antibiotic and preservative resistant opportunistic microorganisms has further complicated the treatment options. It is well known that MK caused by antibiotic-resistant microorganisms are associated with longer hospitalization and poorer visual outcome [8]. There is a great need for an alternative antimicrobial strategy for millions of lens wearers worldwide that may provide broad-spectrum antimicrobial activity bypassing our reliance on preservatives and antibiotic use. 

Ultraviolet light (UV) is part of the electromagnetic spectrum and can be divided into four distinct spectral areas: UVA (wavelengths 315–400 nm); UVB (wavelengths 280–315 nm); UVC (wavelengths 200–280 nm); and vacuum UV (wavelengths 100–200 nm) [9]. Amongst these wavelength ranges, UVC has the highest capacity to inactivate microorganisms because the peak germicidal wavelength is in the range of 250–270 nm and is known as the germicidal spectrum [10]. UVC cause cellular damage by inducing changes in the chemical structure of DNA chains [11]. The consequence is the production of cyclobutene pyrimidine dimers (CPDs) causing distortion of the DNA molecule, which may cause malfunctions in cell replication and lead to cell death [9]. Effect of UVC treatment on sterilization of contact lenses and cases have been reported before [12,13]. UVC has been shown to have high efficacy in killing acanthamoeba cysts following exposure up to 24 minutes [14]. Attempts have been made to incorporate UVC within contact lens disinfection systems which showed statistically significant reduction in microbial load [15,16]. 

UVC irradiation is well known for its germicidal action, however, the use of UVC irradiation for prevention and treatment of localized infections is still in the early stages of development. Previous studies confirmed that UVC inactivation is equally effective to antibiotic-resistant bacteria compared to their native counterparts [17].

This study aimed to investigate the antimicrobial activity of UVC against major microorganisms related to contact lens-related keratitis. A further aim was to determine the potential application of UVC in reducing the microbial contamination of contact lenses and lens cases.

## 2. Results

UVC device showed very high antimicrobial activity against all the microorganisms tested. When tested with contact lenses and lens cases, the UVC device showed a significant reduction in contamination most of the time. 

**Figure 1:**
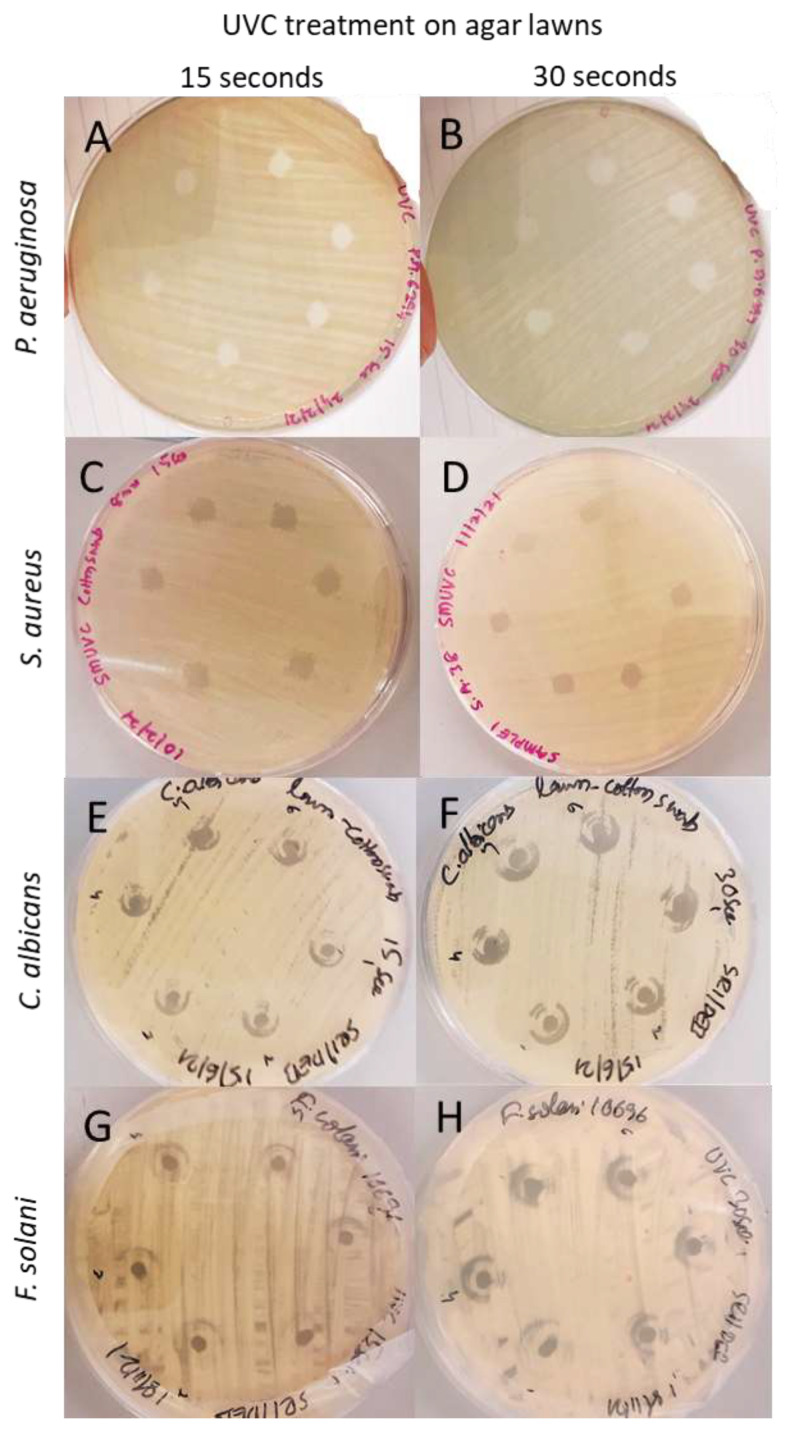
Representative photographs of the agar plates following 15 and 30 s of exposure to UVC device. The photographs demonstrate that 15 and 30 s exposure areas of complete growth inhibition of (**A**,**B**) *P. aeruginosa*, (**C**,**D**) *S. aureus*, (**E**,**F**) *C. albicans*, (**G**,**H**) *F. solani*.

Figure 1 shows inhibition zones of microbial agar lawns following exposure to UVC for 15 and 30 s. The areas exposed to UVC showed inhibition zones, rest of the control areas showed confluent bacterial growth. Both 15 and 30 s exposure were able to fully inhibit microbial growth as identified by the arrow in Figure 1. The diameter of the inhibition zone was slightly increased for 30 s compared to 15 s (Table 1).

The following Table 1 demonstrates that all the microorganisms showed complete inhibition zones, and the area of inhibition varied between the microorganisms tested. *P. aeruginosa* lawns had the largest inhibition zones compared to other microorganisms, whereas *F. solani* had the smallest. Exposure of 30 s had a slightly larger inhibition zone compared to 15 s exposure. This difference was highest with *F. solani* (0.8 ± 0.2 mm), and lowest for *S. aureus* (0.2 ± 0.1 mm).

**Figure 2:**
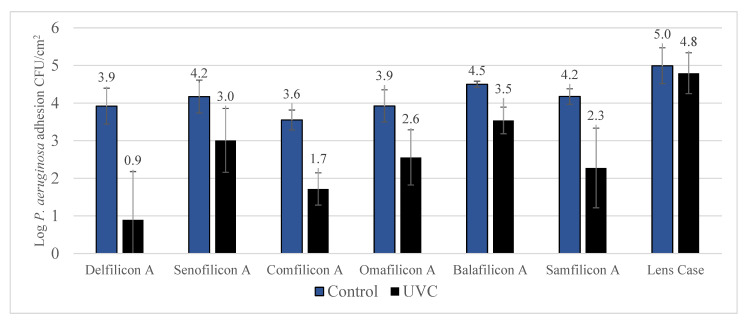
Reduction of *P. aeruginosa* contamination after UVC treatment. Exposure to UVC statistically significant (*p* < 0.001) reduced *P. aeruginosa* contamination of contact lenses. The reduction in contamination observed with lens case showed no significant difference (*p* = 0.194).

The antimicrobial efficacy of UVC treatment on various contact lens materials and lens cases contaminated with *P. aeruginosa* is detailed in Figure 2. Significant (*p* < 0.001) reduction of *P. aeruginosa* contamination was noted following exposure to UVC for all contact lens materials. Reduction of contamination for lens case was 36.2 ± 13.3% (0.28 ± 0.09 log; *p* = 0.194).

**Figure 3:**
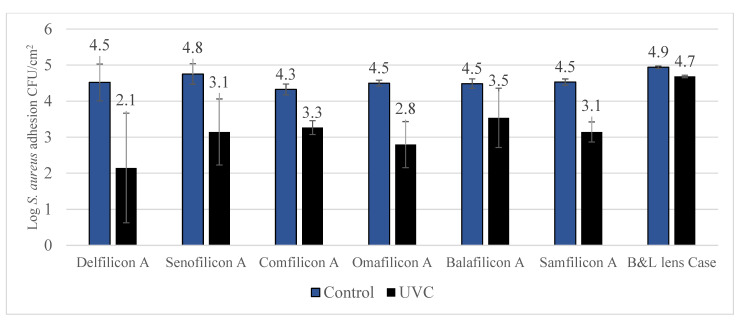
Reduction of *S. aureus* contamination following UVC treatment. Exposure to UVC statistically significant (*p* < 0.001) reduced *S. aureus* contamination of contact lenses and lens cases.

The efficacy of UVC treatment on various contact lens materials and lens cases contaminated with *S. aureus* is detailed in Figure 3. Significant (*p* < 0.001) reduction in contamination was observed against all the tested contact lens materials and the lens case. 

**Figure 4:**
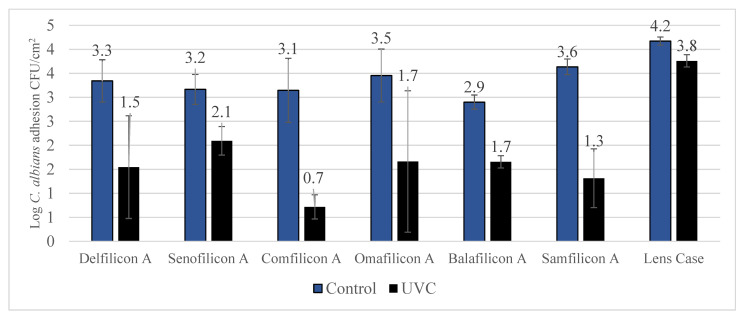
Reduction of *C. albicans* contamination after UVC treatment. Exposure to UVC statistically significant (*p* < 0.001) reduced *C. albicans* contamination of all contact lenses and lens cases.

Reduction of *C. albicans* contamination in contact lenses and lens case following exposure to UVC is detailed in Figure 4. Significant (*p* < 0.001) reduction in contamination was observed against all the tested contact lens materials and the lens case. 

**Figure 5:**
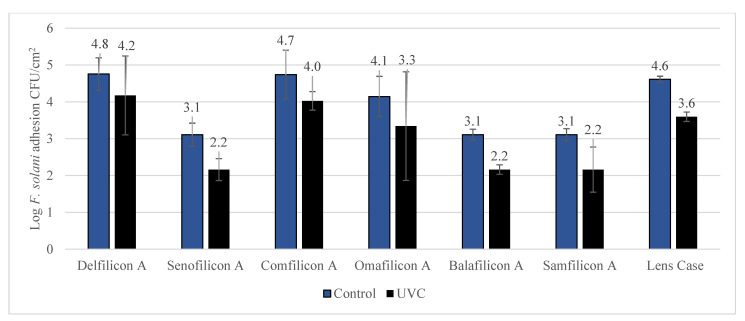
Reduction of *F. solani* contamination after UVC treatment. Exposure to UVC statistically significant (*p* < 0.05) reduced *F. solani* contamination of Delefilcon-A, Senofilcon-A, Comfilcon-A, and Samfilcon-A contact lens materials and lens cases.

The efficacy of UVC exposure to *F. solani* contaminated lenses and lens cases are demonstrated in Figure 5. Overall high efficacy in reduction of contamination was observed for all contact lens materials except for Balafilcon-A (0.55 ± 0.13 log; *p* = 0.189) and Samfilcon-A (0.70 ± 0.26 log; *p* = 0.110). The antimicrobial efficacy with lens case was 90.4 ± 3.3% (1.02 ± 0.39 log) which was statistically significant (*p* = 0.001).

The following Table 2. summarizes the reduction of the percentage of contact lens contamination implicated by UVC treatment. 

## 3. Discussion

The current study found that a very short 15–30 s exposure of UVC can provide high antimicrobial action against most of the predominant microorganisms responsible for contact lens keratitis. In addition, this treatment can substantially reduce contact lens and lens case contamination, with a real potential to reduce these types of keratitis in a clinical setting. 

The UVC device showed total efficacy against *P. aeruginosa*, *S. aureus*, *F. solani*, and *C. albicans* when exposed directly to agar lawns. The 4.5 mm UVC exposure to the microbial lawns showed 5.0 to 5.9 mm inhibition against the fungal strains and 6.9 mm to 7.5 mm inhibition zone against the bacterial strains. Inhibition zones were bigger with bacterial strains compared to fungal strains, which may be because the bacteria at the edges of the inhibition zones were more sensitive to UVC compared to fungal strains, which is supported by the contact lens contamination study where inhibition on bacterial strains was higher compared to fungal strains. The results reported in the current study are slightly higher than previously reported by Dean et al. [18], which showed 3.50 mm to 5.50 mm inhibition zone against bacterial strains, however they did not check against fungal strains. Thai et al. used 254 nm UVC and showed that 180 s of exposure can significantly reduce bacterial load on chronic wounds [19]. Guridi et al. used varying doses (840–3360 mJ/cm^2^) of UVC (253.7 nm) against *P. aeruginosa*, *S. aureus*, and *C. albicans* and found >99.99% efficacy when exposed directly on different biomaterial surfaces [20]. This is in agreement with our results on direct exposure, including on bacteria on contact lens surfaces which often showed >99% reduction in bacterial viability. Umezawa et al. investigated the efficacy of pulsed UVC light (photon peaks spread across 240–400 nm), which showed more than 2 log growth inhibition against similar microorganisms such as *P. aeruginosa* and *S. aureus* [21]. The efficacy of UVC (254 nm) against similar microorganisms on textile surfaces are reported to be more than 90% [22], which is also in line with our reports with high antimicrobial efficacy. 

Contamination of contact lenses and lens cases have been directly implicated in the development of corneal infiltrative events, particularly in various types of keratitis [2,23]. Several antimicrobial strategies have been adopted to reduce contamination of causative microorganisms such as Gram-negative and Gram-positive bacteria and fungal strains [2,24]. Preservatives and disinfectants are the first-line antimicrobial agents used in contact lens care solutions. However more than 50% of lens cases from asymptomatic lens wearers were found to be contaminated, and more than 10% were with opportunistic Gram-negative bacteria [25]. Silver, selenium, Salicylic acid, Fimbrolides and antimicrobial peptides are some of the common strategies that were investigated as additional antimicrobials in the past [2,24,26,27]. The current study indicated that combining UVC treatment with existing care regimes is likely to significantly reduce contamination levels. 

Previous studies have shown that the rate of microbial contamination can significantly vary based on the type of contact lens material used, while 2nd generation silicone hydrogel lenses may attract more microorganisms compared to hydrogel lenses [28]. However, the current study found bacterial contamination to the variety of control lenses are comparable. UVC exposure was able to significantly reduce contamination of both *P. aeruginosa* and *S. aureus* for silicone hydrogel and hydrogel lenses. The inhibition against *P. aeruginosa* ranged between 89% to more than 99% whereas against *S. aureus* inhibition ranged between 88% to 99%. There was no particular pattern found between different types of silicone hydrogel lenses, whereas Delefilcon A lenses were associated with the highest antimicrobial efficacy; more than 2.5 log inhibition with UVC against *P. aeruginosa*. Similar results were observed with *S. aureus*. 

Depending on the geographical location, contact lens wear is often the most common risk factor for the development of fungal keratitis. This can often exceed 50% of the cases. Fusarium and Candida are the most common types of fungal strains implicated in contact lens-related keratitis, isolated from 41% and 14% of culture-positive tests [29]. The current study showed that UVC irradiation can significantly reduce *C. albicans* contamination ranging between 1.07 to 2.43 log inhibition based on the type of contact lens material used. A similar trend was observed against *F. solani*, where UVC showed inhibition ranging between 0.90 to 0.71 log. Although UVC showed 71% and 79% inhibition against *F. solani* in Balafilcon-A and Samfilcon-A lens materials, the differences were not statistically significant. The current study is one of the few studies that has used Balafilcon-A and it is the first study to have used Samfilcon-A for investigation with fungal strains, thus requiring further investigation with these lens materials. The overall, high antifungal efficacy of UVC irradiation coupled with the existing contact lens care regimen would certainly provide high and comprehensive fungicidal activity, protective towards contact lens-related fungal keratitis. 

This study found that microbial contamination of contact lens cases was higher compared to lenses, which is likely due to the formation of biofilms of lens cases. Various enzymes, antibiofilm peptides, and other dispersion molecules have been investigated for medical-biofilm dispersion [30]. A limited number of agents including antimicrobial peptides, furanones, silver and passive dispersion agents have been tested on contact lens cases [2]. Contact lens cases are known to harbour microbial biofilms and have been directly associated with keratitis events [31]. The current study showed that UVC irradiation can significantly reduce *S.aureus* (44%), *C. albicans* (61%) and *F. solani* (90%), however, only 36% inhibition against *P. aeruginosa* was achieved. 

This study did not investigate the safety of the UVC which was reported earlier [18]. Dean et al. reported that up to 30 s of exposure to UVC did not stimulate the death of human corneal epithelium [18]. Although the current study did not expose human cells to UVC, it is important to note that UVC has very little penetration on the cornea and is unlikely to impact the corneal endothelium. UV rays are known to cause photokeratitis which is also called ultraviolet photokeratitis. However, the ocular tissue damage threshold for UV rays is 5 mJ/cm^2^, and the LED used for irradiation in this study emits less than 2 mJ/cm^2^. Given that the UVC exposure is aimed to decontaminate contact lenses and lens cases, accidental exposure to the eye is unlikely to cause any major harm. This study did not examine any detrimental effect of direct UVC exposure to contact lenses. Polymerization of contact lens monomer include exposure to UVC, hence we assumed that the short exposure of UVC to contact lens materials unlikely to have any significant change in the key parameters such as base curve, diameter, refractive index and oxygen transmissibility. 

## 4. Materials and Methods

### 4.1. Ultraviolet C device:

**Figure 6:**
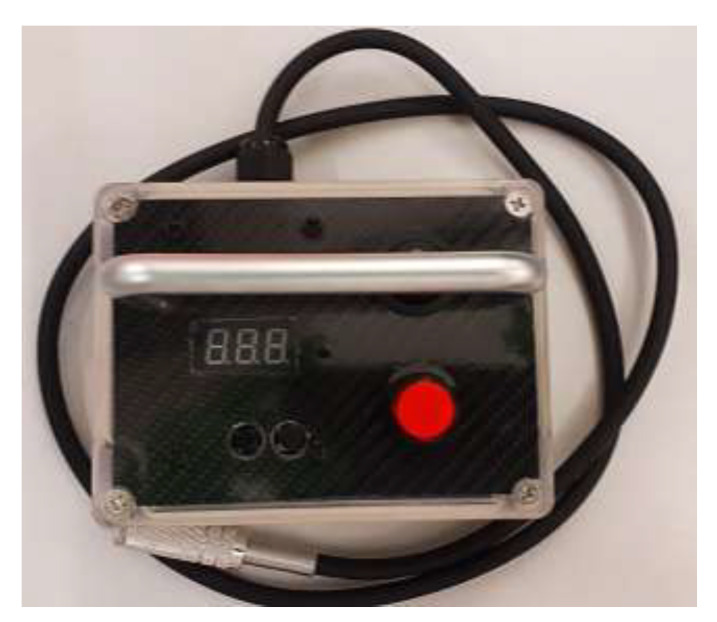
UVC device in this study.

The prototype device comprises a 265-nm (Figure 6) (Photon Therapeutics; Oldsmar, UK) detailed earlier [18]. Briefly, it contains a hemispheric ball lens, which is protected by a rubber sheath 8 mm length, projecting a spot size of 4.5 mm, resulting in an intensity of 1.93 mJ/cm^2^ at the target distance, as confirmed with a calibrated UVC light meter (Solar meter Model 8.0 UVC, Solartech Inc, Harrison Twp, MI, USA). Power was supplied by a 9 V DC regulated adapter with an additional current limiting circuit [18]. 

Bacterial lawns were freshly prepared on Nutrient Agar (NA; Sigma Aldrich, St. louis, MO, USA) and fungal lawns were made on Potato Dextrose Agar (PDA: Merck Ga A, Damstadt, Germany) plates from the previously prepared suspensions. The plates were exposed to 4.5 mm diameter UVC beam for 15 and 30 s at an 8 mm distance. After 24 h incubation at 37 °C for bacteria or 2 days incubation at 37 °C for *C. albicans* and 4 days incubation at 25 °C for *F. solani*, the efficacy of the UVC beam was examined by investigating the diameter of the treatment zone, using a digital colony counter (Stuart Company, London, UK). A total of three horizontal and three vertical measurement of the inhibition zone were made and the average and standard deviation was reported. 

### 4.2. Contact lenses and Lens cases

Widely used and most popular contact lenses were used in this study, their parameters, materials and other properties are described in Table 3. Bausch and Lomb contact lens cases (Bausch and Lomb UK Ltd., Kingston, UK) were used in this study. 

### 4.3. Strains and microbial conditions 

*Pseudomonas aeruginosa* strain 6294 and *Staphylococcus aureus* strain 38 isolated from MK cases were used in this study. *Fusarium solani* ATCC 10696 isolated from soil and *Candida albicans ATCC 76615*, a clinical isolate were used in this study. Bacteria were grown overnight in TSB (Melfold, UK) at 37 °C with aeration. The harvested bacterial cells were centrifuged for 10 minutes at 3000 rpm and the cells were washed three times with phosphate-buffered saline (PBS; pH 7.4; NaCl 8 g L^−1^, KCl 0.2 g L^−1^, Na_2_HPO_4_ 1.15 g L^−1^, KH_2_PO_4_ 0.2 g L^−1^). *P. aeruginosa* were then resuspended in PBS and *S. aureus* were resuspended in 10% TSB to an OD_660nm_ of 0.1 (1 × 10^8^ CFU mL^−1^). The bacterial cell suspensions were then diluted to 1 × 10^6^ CFU mL^−1^. *C. albicans* strains were grown on PDA plates by incubating for 24 h at 37 °C, then suspended in sterile PBS to an OD_660nm_ of 1.5 (1 × 10^8^ CFU mL^−1^) and the suspensions were serially diluted to 1.0 × 10^6^ CFU mL^−1^ and used for adhesion assays. *F. solani* were grown on PDA plates by incubating for 7 to 10 days at 25 °C followed by filtering through sterile 70 µm filters to remove hyphal fragments and finally resuspended to an OD_660nm_ of 2.6 (1 × 10^8^ CFU mL^−1^).

Microbial assays with contact lenses and lens cases have been detailed earlier [32]. Briefly, contact lenses were washed two times in PBS and transferred to 1ml of bacterial or fungal suspensions in wells of 24-well tissue culture plates (CELESTAR®, Greiner bio-one, Frickenhausen, Germany), keeping concave side up. To allow contamination, lenses were incubated with 1mL bacterial suspension for 18 h at 37 °C and for fungal strains 18 h at 25 °C with shaking (120 rpm). Lens cases were incubated similarly but with 2 mL microbial suspensions in the lens case cup. After this, lenses were aseptically removed from the microbial suspensions and washed twice with 1ml PBS in a 24 well plate by shaking at 120 rpm for 30 s to remove non-adherent cells. Lens cases were washed with 1 mL PBS twice by shaking 120 rpm for 30 s. 

Following exposure to microorganisms, each contact lens was cut into equal 4 samples with a sterile scalpel, one piece used as control and the rest three pieces were placed 8 mm beneath a 265 nm UVC lamp for 30 s. Four 4 mm non-overlapping UVC beams were exposed to both sides of the lens. Similarly, each lens case was exposed to 9 non-overlapping 30 s spots. 

After this, all lens samples were placed in a 2 mL sterile plastic vial containing 2 mL PBS with a sterile magnetic bar and vortexed for at least one minute. Control and UVC-exposed lens cases were filled with 2 mL PBS and a sterile magnetic bar and vortexed for at least one minute. For bacterial and *C. albicans* strains, following log serial dilutions in PBS, three 50 micro-litre droplets of each dilution were plated on NA and PDA plates for recovery of cells respectively. For *F. solani*, following log serial dilutions in PBS, 100 micro-litre were plated onto PDA for recovery of viable cells. After 24 h incubation at 37 °C for bacteria or 2 days incubation at 37 °C for *C. albicans* and 4 days incubation at 25 °C for *F. solani*, the viable micro-organisms were enumerated as colony-forming units (CFU). Results are expressed as the reduction in viable bacteria or fungi (compared with the untreated control samples). Three samples were used for each experiment and were repeated for at least three separate occasions.

The adhesion data were log_10_ (x + 1) transformed prior to data analysis where x is the adherent bacteria or fungi in colony-forming units. The reuction of adhesion data was presented as mean ± standard deviation. Differences in the microbial load were analyzed using the Wilcoxon-Signed ranked test. Differences between the groups were analyzed using linear mixed model ANOVA, which adjusts the correlation due to repeated observations. Post hoc multiple comparisons were done using Bonferroni correction. Statistical significance was set at 5%.

## 5. Conclusions

In conclusion, this study showed that the ophthalmic device with a very short UVC exposure has potent antimicrobial activity against a majority of the causative microorganisms for contact lens-related keratitis. The device is particularly effective in reducing contamination on contact lenses and lens cases.. This study further demonstrate that UVC could be readily used as a preventative measure and inhibition of broad-spectrum antimicrobial contamination.

## Figures and Tables

**Table 1:** Inhibition zone diameter (mm) following UVC exposure.

Microorganisms	15 s Exposure	30 s Exposure
*P. aeruginosa* 6294	7.2 ± 0.3	7.5 ± 0.4
*S. aureus* 38	6.9 ± 0.3	7.1 ± 0.4
*C. albicans* ATCC 76615	5.5 ± 0.4	5.9 ± 0.2
*F. solani* ATCC 10696	5.0 ± 0.4	5.8 ± 0.5

**Table 2:** Percent of reduction in contact lens microbial contamination (mean ± SD) following UVC treatment. Asterix (*) indicates statistically significant difference.

Contact Lenses	*P. aeruginosa*	*S. aureus*	*C. albicans*	*F. solani*
Delefilcon A	99.9 ± 5.2 *	99.6 ± 10.3 *	98.4 ± 26.7 *	76.0 ± 5.3 *
Senofilcon A	93.2 ± 4.3 *	97.5 ± 8.9 *	91.5 ± 13.3 *	68.3 ± 7.3 *
Comfilcon A	98.5 ± 14.3 *	91.2 ± 5.8 *	99.6 ± 4.2 *	80.5 ± 13.1 *
Omafilcon A	97.6 ± 15.5 *	98.0 ± 8.5 *	98.4 ± 17.8 *	73.7 ± 8.6 *
Balafilcon A	89.0 ± 7.8 *	88.8 ± 13.3 *	94.3 ± 3.4 *	71.5 ± 10.3
Samfilcon A	98.7 ± 7.1 *	95.9 ± 7.8 *	99.5 ± 10.3 *	79.9 ± 26.3
Lens Case	36.7 ± 13.3	44.7 ± 12.6 *	61.2 ± 3.4 *	90.4 ± 3.3 *

**Table 3:** Properties of contact lens materials used in the study.

Proprietary Name	Total Dailes1	Acuvue Oasys	Biofinity	Proclear	Purevision2	Ultra
United States Adopted Name (USAN)	Delfilicon A	Senofilicon A	Comfilicon A	Omafilicon A	Balafilicon A	Samfilicon A
Lens material	Silicone hydrogel	Silicone hydrogel	Silicone hydrogel	Hydrogel	Silicone Hydrogel	Silicone Hydrogel
Manufacturer	Alcon	Johnson & Johnson	Cooper vision	Cooper vision	Bausch & Lomb	Bausch & Lomb
Water content (%)	Gradient	38	48	62	36	46
Oxygen transmissibility (DK/t)	156	147	160	37	130	163
Centre thickness (mm) -3.00DS	0.09 mm	0.07 mm	0.08 mm	0.09 mm	0.07 mm	0.07 mm

## Data Availability

This study did not report any data.
